# A potent synthetic inorganic antibiotic with activity against drug-resistant pathogens

**DOI:** 10.1038/srep41999

**Published:** 2017-02-06

**Authors:** Shelby Hubick, Arumugam Jayaraman, Alexander McKeen, Shelby Reid, Jane Alcorn, John Stavrinides, Brian T. Sterenberg

**Affiliations:** 1 Department of Biology, University of Regina, 3737 Wascana Parkway, Regina, Saskatchewan, S4S0A2, Canada; 2 Department of Chemistry and Biochemistry, University of Regina, 3737 Wascana Parkway, Regina, Saskatchewan, S4S0A2, Canada; 3 College of Pharmacy and Nutrition, University of Saskatchewan, 104 Clinic Place, Saskatoon, Saskatchewan, S7N2Z4, Canada

## Abstract

The acronymously named “ESKAPE” pathogens represent a group of bacteria that continue to pose a serious threat to human health, not only due to their propensity for repeated emergence, but also due to their ability to “eskape” antibiotic treatment^1^,^2^. The evolution of multi-drug resistance in these pathogens alone has greatly outpaced the development of new therapeutics, necessitating an alternative strategy for antibiotic development that considers the evolutionary mechanisms driving antibiotic resistance. In this study, we synthesize a novel inorganic antibiotic, phosphopyricin, which has antibiotic activity against the Gram-positive methicillin-resistant *Staphylococcus aureus* (MRSA) and vancomycin-resistant *Enterococcus faecium* (VRE). We show that this potent antibiotic is bactericidal, and exhibits low toxicity in an acute dose assay in mice. As a synthetic compound that does not occur naturally, phosphopyricin would be evolutionarily foreign to microbes, thereby slowing the evolution of resistance. In addition, it loses antibiotic activity upon exposure to light, meaning that the active antibiotic will not accumulate in the general environment where strong selective pressures imposed by antibiotic residuals are known to accelerate resistance. Phosphopyricin represents an innovation in antimicrobials, having a synthetic core, and a photosensitive chemical architecture that would reduce accumulation in the environment.

Widespread and increasing antibiotic resistance among the “ESKAPE” pathogens (*Enterococcus faecium, Staphylococcus aureus, Klebsiella pneumoniae, Acinetobacter baumannii, Pseudomonas aeruginosa*, and *Enterobacter spp.*)^1^ has become a critical problem for healthcare. Organic natural products, which have provided the core set of current therapeutics, have been central to our ability to control these multi-drug resistant pathogens; however, microbes have been harnessing antibiotics for competition for billions of years, and as a result, mechanisms for resisting and tolerating antibiotics have been evolving for just as long^3^,^4^. For each new antimicrobial natural product discovered, at least one mechanism of resistance is already present in the general environment, greatly accelerating the emergence of antibiotic resistance^5^. In fact, over evolutionary time, microbes have likely encountered most naturally occurring organic and inorganic molecules in the environment, and have evolved specific strategies to contend with those that are immediately toxic^3^. Compounding the antibiotic resistance problem are the high concentrations of anthropogenically-generated antibiotic residuals that are deposited into the environment, resulting in strong selective pressures for the evolution of resistance^6^,^7^.

One possible strategy for overcoming these obstacles and regaining some ground on the antibiotic resistance problem is to develop synthetic antimicrobials whose chemical architectures do not occur naturally, and would thus be evolutionarily foreign to bacteria. In addition, if such antimicrobials degraded rapidly in the general environment, strong selective pressure for the evolution of resistance would be reduced significantly. Organophosphorus compounds remain an untapped pool of chemical architectures that could possess novel chemistries and differential binding affinities to diverse microbiological targets. Also, because phosphorus has a similar electronegativity to carbon, the chemistry of low valent phosphorus often resembles that of carbon, making it highly amenable for syntheses of compounds for biological applications^8^. Phosphorus-containing antibiotics have been synthesized previously, including fosfomycin^9^, clindamycin^10^, and torezolid^11^; however, these molecules are organophosphates that may be susceptible to existing antibiotic resistance mechanisms.

In contrast to organophosphates, phosphine derivatives remain underexplored as potential antimicrobials^12^,^13^. These compounds provide a large pool of potentially biologically active molecules with vast structural diversity, mainly due to the ease with which they can be modified chemically^14^. Phosphines also provide a simple means of introducing metal complexes into biologically active molecules, via coordination of the lone pair, further expanding the range of inorganic groups that can be explored. Phosphine derivatives have been shown to have potential as anti-cancer drugs^13^, and even to have potential as antibiotics^12^. Some have been developed into resistance protein inhibitors, including avibactam, clavulanic acid, tazobactam, and sulbactam^15^,^16^,^17^,^18^. One recent study synthesized phosphine derivatives of ciprofloxacin and norfloxacin, yielding an additional class of broad-spectrum antibiotics effective against *P. aeruginosa, S. aureus, K. pneumoniae*, and *E. coli*^19^. Phosphorus-containing functional groups could yield compounds with unique biochemistry, such as stronger binding affinity to certain enzymes as compared to carbon or nitrogen analogs. Non-phosphate, organophosphorus compounds therefore have enormous capacity to provide new chemical architectures for the development of next-generation antibiotics.

In this study, we generated a library of synthetic organophosphorous compounds, and surveyed for antimicrobial activity. We identified one compound that has antibiotic activity against the Gram-positive methicillin-resistant *Staphylococcus aureus* (MRSA) and vancomycin-resistant *Enterococcus faecium* (VRE), and subsequently carried out systematic replacement of its chemical side groups to increase its potency. We show that the resulting compound, called phosphopyricin, is bactericidal, exhibits low toxicity in mice, and loses activity when exposed to light.

## Results and Discussion

**Figure 1:**
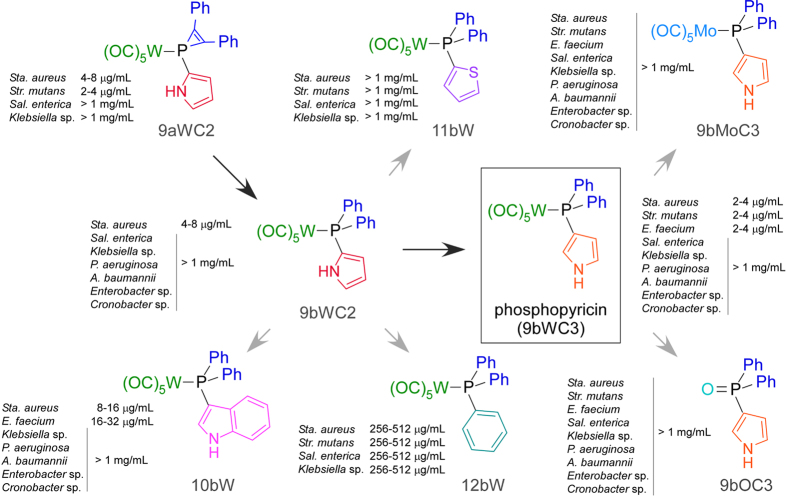
Development of phosphopyricin (9bWC3) by derivatization. Elimination of the phosphirene ring of antimicrobial compound 9aWC2 yielded compound 9bWC2, both of which had similar minimum inhibitory concentrations against *Staphylococcus aureus* K1-7. Substitution of the C2-bonded pyrrole of 9bWC2 with a thiophene, phenyl or indole reduced activity. Replacement of the C2-bonded pyrrole of 9bWC2 with a C3-bonded pyrrole resulted in phosphopyricin (9bWC3), and increased potency against *S. aureus*. Phosphopyricin had similar activity against the Gram-positive *Enterococcus faecium* K02G0810 and *S. mutans* UAIS9:wt. Substitution of the tungsten pentacarbonyl group of phosphopyricin with either a molybdenum pentacarbonyl group or an oxide abolished activity (<1024 μg/mL).

**Figure 2:**
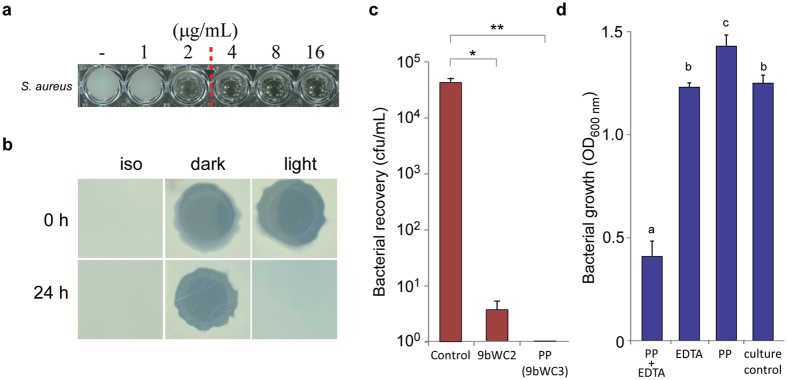
Properties of phosphopyricin. (**a**) Minimum inhibitory concentration of phosphopyricin against *Staphylococcus aureus* K1-7. (**b**) Activity of phosphopyricin against *S. aureus* K1-7 following exposure to 24 hours of light, as compared to dark and isopropanol (iso) controls. (**c**) Recovery of *S. aureus* K1-7 from culture exposed to either phosphopyricin (PP) or 9bWC2, relative to the antibiotic-free control. *p < 10^−5^; **p < 10^−6^. (**d**) Optical density of *S. enterica* incubated in the presence of either phosphopyricin with EDTA (PP + EDTA), EDTA alone (EDTA), phosphopyricin alone (PP), or with neither (culture control). Letters above error bars (s.d.) indicate statistically significantly different groups (p < 0.005). Cropped images in panel (**b**) were taken from the same plate for each respective time point. No image enhancements were made to these images (including brightness or contrast).

We used metal-mediated electrophilic substitution^20^,^21^,^22^ to rapidly generate a diverse initial library of synthetic organophosphorus compounds containing carbon-phosphorus bonds. One compound in the library, 9aWC2, a tungsten phosphine complex (Fig. 1), showed inhibitory activity against *Staphylococcus aureus* K1-7 at a minimum inhibitory concentration (MIC) of 4–8 μg/mL. This promising candidate antimicrobial was modified to eliminate the synthetically challenging phosphirene ring, yielding compound 9bWC2, which exhibited similar potency towards *S. aureus* (MIC of 4–8 μg/mL) (Fig. 1). To identify features of 9bWC2 that were essential for antimicrobial activity, we systematically varied chemical side groups, beginning with substitution of the C2-bound pyrrole with C2-bound thiophene, which resulted in loss of antimicrobial activity against *S. aureus* (11bW, MIC > 1024 μg/mL) (Fig. 1). Substitution of pyrrole with indole resulted in slightly reduced potency against *S. aureus* (10bW, MIC of 8–16 μg/mL), while substitution of the pyrrole with a phenyl group, significantly reduced potency against *S. aureus* (12bW, 256–512 μg/mL), but increased the spectrum of activity to include Gram-negative, *Klebsiella* sp. and *Salmonella* sp. (MIC of 256–512 μg/mL) (Fig. 1). We then replaced the C2-bound pyrrole ring with a C3-bound pyrrole (9bWC3), resulting in an increased potency against *S. aureus*, from a MIC of 4–8 μg/ml to 2–4 μg/ml (Fig. 2A). An evaluation of the potency of this compound against the Gram-positive *Enterococcus faecium* K02G0810 and *S. mutans* UAIS9:wt revealed that both were also inhibited at MICs of 2–4 μg/mL, and spot testing on bacterial overlay plates containing all three Gram-positive bacteria revealed no spontaneously resistant mutants. This compound, 9bWC3, was named phosphopyricin (Fig. 1). Substitution of the tungsten pentacarbonyl group of phosphopyricin with molybdenum pentacarbonyl (9bMo) or oxygen (9bO) abolished detectable antibiotic activity (below 1024 μg/mL), suggesting an important role for the W(CO)_5_ fragment, and the P-bound N-heterocycle.

We observed two important properties of 9bWC2 and phosphopyricin (9bWC3). Firstly, we noted that potency of 9bWC2 decreased significantly upon exposure to light. After 4 hours of fluorescent light exposure at 92.7 μmol/s. m^2^, the MIC of 9bWC2 for *S. aureus* K1–7 increased from 4–8 μg/mL to 8–16 μg/mL, suggesting photolytic degradation. Like its parent compound, phosphopyricin activity was also markedly reduced after 24 hours in continuous fluorescent light at the same intensity (Fig. 2B), suggesting that phosphopyricin would photodegrade gradually in the general environment. Secondly, we determined that both 9bWC2 and phosphopyricin were bactericidal. Bacteriostatic agents are able to arrest bacterial growth, while bactericidal agents compromise bacterial cell viability and eradicate >99.9% of a given inoculum^23^. Approximately 5 × 10^5^ cfu of *S. aureus* K1-7 was exposed to either 50 μg/mL phosphopyricin or 9bWC2 for 24 hours in 1 mL 10 mM MgSO_4_ buffer, and the viability of the bacteria was subsequently assessed. Relative to the antibiotic-free control, viable cell number was reduced by 99.9993% for 9bWC2, and 100% for phosphopyricin (Fig. 2C). Given this potency, we assessed its activity against the Gram-negative *Pseudomonas aeruginosa, S. enterica* subsp. Typhimurium 14028, *Cronobacter sp.* 12202, *Enterobacter* sp. TX1, *Klebsiella* sp. B011499, and *Acinetobacter baummanii* ATCC17978, and all were shown to be resistant (>1024 μg/mL). The outer membrane of Gram-negative strains can act as a barrier to foreign agents, including antimicrobials, but can be destabilized with compounds like the chelating agent, ethylenediaminetetraacetic acid (EDTA)^24^,^25^. For example, the Gram-negative *S. enterica* subsp. Typhimurium 14028 becomes sensitive to the Gram-positive-specific antibiotic, nisin, in the presence of EDTA, which destabilizes the outer membrane, thereby overcoming physical exclusion of the antibiotic from the cell^24^. We incubated *S. enterica* subsp. Typhimurium 14028 for 24 hours at 37 °C in the presence of 32 μg/mL phosphopyricin, with and without 1.5 mM EDTA. Bacterial titres were reduced significantly when EDTA was combined with phosphopyricin, as compared to cultures containing only phosphopyricin (Tukey’s HSD, p < 2 × 10^−12^) (Fig. 2D). This suggests that the target(s) of phosphopyricin are also present in Gram-negative bacteria, and that the spectrum of activity is determined by the ability of the compound to cross the bacterial cell wall. Thus, the spectrum of activity of phosphopyricin can be expanded with additives that increase bacterial cell wall permeability.

Although phosphopyricin is a potent antimicrobial, it contains a transition metal, tungsten, which could bind competitively to essential animal proteins where other co-factors, like molybdenum cofactor, would normally bind^26^. Because of this, we evaluated the potential toxicity of phosphopyricin over 14 days in 9 week-old female mice (n = 5) using a single oral dose of up to 400 mg/kg phosphopyricin in a 1% methylcellulose/1% Tween80 vehicle. No mice died during the observation period, and there were no statistically significant differences in food intake, water intake, or body weight changes between control mice and those receiving up to 400 mg/kg of phosphopyricin (Mann-Whitney, p > 0.05). No observable changes in behaviour or other clinical signs were noted, and necropsies showed no evidence of damage to brain, lung, kidneys, gastrointestinal tract, heart, liver, or reproductive tract. Several other studies have shown that oral and dermal exposure to tungsten results in rapid absorption and distribution throughout the body with no major toxic effects^26^,^27^,^28^,^29^. For example, oral exposure assays of mice and rats revealed elevated levels of tungsten in liver, kidney, uterus, femur and intestine that peaked at one and four hours, respectively, but levels in all examined tissues returned to baseline after 24 hours^27^,^28^. Similarly, other studies conducting inhalation exposure assays in rats revealed high levels of tungsten in brain tissue; however, these levels returned to baseline after three days indicating that tungsten does not persist in the body^26^,^30^. Although these and many other studies suggest relatively low toxicity, it is important to note that the water-soluble tungstate salts or the more insoluble tungsten oxides and carbides that they used may have different pharmacokinetics than compounds like phosphopyricin, which contains a tungsten pentacarbonyl group.

Synthetic organic antibiotics based on conserved cores have expanded the pool of effective antibiotics in the short term^31^, yet their inherent common structure has enabled microbes to acquire new resistance mechanisms that target the common backbone, or to evolve resistance through incremental mutations of existing genetic determinants^32^,^33^. Here we demonstrate that organophosphorus compounds, a group that has not been evaluated for antimicrobial activity in any systematic fashion, have the capacity to yield new architectures for clinically relevant pathogens. In addition, non-phosphate organophosphorus compounds have the incredible advantage over existing antibiotics in that these compounds do not occur naturally in the environment, such that resistance to these foreign compounds would be less likely to have already evolved. Although microbes have been shown to possess specific strategies for utilizing, metabolizing, or simply contending with phosphate compounds in the environment^34^, the evolution of resistance to these synthetic organophosphorus compounds may be slowed considerably, since microbes are less likely to be able to metabolize these xenobiotics^35^,^36^. The exploration of synthetic organophosphorus compounds has the potential to make a significant impact for antimicrobial discovery, and to move the field in a new direction that considers not only molecular mechanisms of resistance, but also the evolutionary pressures that drive it.

## Methods

### Bacterial isolates and culturing conditions

*Staphylococcus aureus* K1-7, *Pseudomonas aeruginosa* ATCC 27853*, Enterococcus faecium* K02G0810, *Cronobacter* sp. 12202, *Enterobacter* sp. TX1, *Klebsiella* sp. B011499, *Klebsiella* sp. G4061350*, Acinetobacter baumannii* ATCC17978, and *Streptococcus mutans* UAIS9:wt were cultured in lysogeny broth (LB, BD Biosciences) at 37 °C, except for *E. faecium* K02G0810, which was cultured in brain-heart infusion (BHI, BD Biosciences) medium at 37 °C.

### Chemical synthesis and derivatization

Compounds 9aWC2, 9bWC2, 9bWC3, 10bW, and 11bW were synthesized using published procedures^20^,^21^,^22^. 9bWC2 and 9bWC3 are isomers that result from C2 or C3 substitution of the phosphine on the pyrrole ring, and were separated for the bioassays as described previously^12^. 9bWC3 was given the name, phosphopyricin. ‘Phospho’ is derived from phosphorus, ‘pyr’ is derived from pyrrole and ‘icin’ is a common ending used for names of several classes of antibiotics, and is used here to indicate that this compound has antimicrobial properties. The IUPAC name for phosphopyricin (9bWC3) is pentacarbonyl(diphenyl(3-pyrrole)phosphane)tungsten.

To synthesize O = PPh_2_(C_4_H_3_NH) (9bO), compound [W(CO)_5_{PPh_2_(C_4_H_3_NH)}] (9bW, 60 mg, 0.104 mmol, 2 isomers) and dppe (43.6 mg, 0.110 mmol) were dissolved in THF (2 mL) and irradiated with UV for 2 h, resulting in a color change from colorless to yellow. The solvent was removed under reduced pressure, and the residue was purified by flash chromatography (silica gel, 50/50 v/v diethyl ether/petroleum ether). During workup in air, the free phosphine oxidizes to the phosphine oxide. The phosphine oxide was isolated as a mixture of two isomers, O = PPh_2_(2-C_4_H_3_NH) (9bOC2) and O = PPh_2_(3-C_4_H_3_NH) (9bOC3 in a 20:80 ratio. Because this isomer mixture showed no antibiotic activity, the isomers were not separated and characterized. Yield: 17 mg, 62%. ^31^P{^1^H} NMR (CDCl_3_): *δ* 19.3 [O = PPh_2_(2-C_4_H_3_NH)] and 22.3 [O = PPh_2_(3-C_4_H_3_NH)]. ^1^H NMR (CDCl_3_): 6.36 (m, 2H, pyrrole 3-H), 7.05 (m, 1H, pyrrole 2-H), 7.33–7.60 (m, Ph), 9.30 (br, 1H, NH) [O = PPh_2_(2-C_4_H_3_NH)]; 6.29 (m, 1H, pyrrole 3-H), 6.80 (m, 1H, pyrrole 2-H), 6.92 (m, 1H, pyrrole 2-H), 7.33–7.60 (m, Ph), 9.30 (br, 1H, NH) [O = PPh_2_(3-C_4_H_3_NH)].

The synthesis of [W(CO)_5_{PPh_3_}] (12bW) was carried out by first adding (CH_3_)_3_NO·2H_2_O (66.3 mg, 0.597 mmol) to a solution of [W(CO)_6_] (200 mg, 0.568 mmol) in acetonitrile (15 mL), in small portions, over 5 min. The resulting yellow solution was stirred for 40 min, and the solvent was removed under reduced pressure. The residue was dissolved in toluene (2 mL) and the solvent was again removed under reduced pressure. The crude [W(CO)_5_{CH_3_CN}] was dissolved in THF (15 mL), PPh_3_ (164 mg, 0.625 mmol) was added, and the mixture was heated at 50 °C for 16 h. The solvent was removed under reduced pressure, and the residue was purified by flash chromatography (silica gel, 10/90 diethyl ether/petroleum ether). The white product was crystallized by cooling a saturated hexane/diethylether solution to −20 °C. Yield: 35%. IR (*ν*CO, CH_2_Cl_2_, cm^−1^): 2071(w), 1944(vs). ^31^P{^1^H} NMR (CDCl_3_): *δ* 21.6 (^1^*J*_PW_ = 244 Hz). These spectral data match published data for this compound^37^.

To synthesize [Mo(CO)_5_{PPh_2_(C_4_H_3_NH)}] (9bMo), a solution of Mo(CO)_6_ (2.00 g, 7.58 mmol) and PPh_2_Cl (1.36 mL, 9.09 mmol) in dry toluene (50 mL) was heated under reflux for 90 minutes resulting in a colour change from yellow to amber colour. The volume was reduced under vacuum to ~10 mL, and 10 mL of petroleum ether was added. The solution was filtered and anhydrous alumina (1 g) was added, the mixture was shaken and then filtered. The filtrate was evacuated to yield [Mo(CO)_5_(PPh_2_Cl)] as a pale yellow powder. Yield: 2.25 g (65%). ^31^P{^1^H} NMR = *δ* 123, IR (*ν*CO, CH_2_Cl_2_, cm^−1^): 2081, 1969, 2020 cm^−1^. Silver trifluoromethanesulfonate (90 mg, 0.350 mmol) was added to a solution of [Mo(CO)_5_(PPh_2_Cl)] (80 mg, 0.175 mmol) in CH_2_Cl_2_ (2 mL). The solution was stirred for 3 hours, and then filtered to remove AgCl. Pyrrole (24 μL, 0.350 mmol) was added, the solution was stirred for 5 minutes and then the solvent was evaporated under reduced pressure. The residue was purified using column chromatography (silica gel, 10:90 v/v diethyl ether/petroleum ether) to yield 30 mg of a white powder, which was shown to be a mixture of [Mo(CO)_5_(PPh_2_(3-C_4_H_3_NH)] and [Mo(CO)_5_(PPh_2_(3-C_4_H_3_NH)] in a 35:65 ratio. Yield: 37%. ^31^P{^1^H} NMR (CDCl_3_): *δ* 15.8, and 15.

Because the isomer mixture showed no antibacterial activity, the isomers were not separated and characterized. For bioassays, powdered organophosphorus compounds were resuspended in either 100% isopropanol (9aWC2, 9bWC2, 9bWC3, 9bOC3, 10bW), dimethylsulfoxide (11bW) or a 50:50 isopropanol-tetrahydrofuran (v/v) solution (9bMoC3).

### Minimum inhibitory concentration and bactericidal assays

The minimum inhibitory concentrations (MIC) of each compound was assessed in 96-well plates by serial dilution of a 10 mg/mL stock solution of the organophosphorus compound in LB or BHI medium from 1 μg/mL to 1024 μg/mL (one per column). A no-compound (solvent-only) control was used in the final column of the 12-column plate. Approximately 1 × 10^5^ cfu of bacteria was added to each well to a final volume of 200 μL, the microplate covered with sterile, breathable rayon film (VWR International), and incubated at 37 °C with shaking at 220 RPM for 24 hours.

To evaluate bacteriostatic or bactericidal activity, an overnight culture of *S. aureus* K1-7 in LB medium was diluted to yield 1 mL suspensions of 5 × 10^5^ cfu in 10 mM MgSO_4_. Each suspension was treated with a final concentration of 50 μg/mL of either 9bWC2 or 9bWC3 (in isopropanol). Control treatments were supplemented with an equal volume of isopropanol. Cultures were shaken at 220 RPM for 24 hours at 21 °C, and bacterial survivorship assessed by colony enumeration of serial dilutions plated on LB.

### EDTA Assay

To evaluate the effects of phosphopyricin on Gram-negative bacteria, we adapted the protocol of Stevens *et al*.^24^. *S. enterica* subsp. Typhimurium 14028 was cultured in BHI broth at 37 °C overnight, and 1 mL of bacterial culture (OD_600nm_ = ~1.25) was transferred to a 1.5 mL microfuge tube and bacteria pelleted by centrifugation (1 minute at 12.0 × 1000 min^−1^ × g). The supernatant was removed, and cells were re-suspended in 1 mL 10 mM MgSO_4_. Using a 96-well plate, 1 × 10^4^ cfu were incubated in BHI broth containing 1.5 mM EDTA solution, 32 μg/mL phosphopyricin, or both at 37 °C for 24 hours. *S. enterica* subsp Typhimurium 14028 cells grown in BHI broth alone served as a no-treatment control.

### Photolysis assay

Glass vials containing compound stock solution of 9bWC3 in isopropanol at a concentration of 10 mg/mL were placed in a light chamber at 21 °C, and subjected to either 8, 16 or 24 hours of 92.7 μmol/s.m^2^ continuous fluorescent light generated by three, 60 cm Philips F20T12/CW, 20-watt bulbs. The amount of light was measured with a Li-cor Quantum/Radiometer/Photometer (LI-189). Control vials were wrapped in aluminum foil and placed in the same conditions. Following treatment, compounds were spotted on overlay media plates containing *S. aureus* K1-7, as prepared previously^38^.

### Mouse toxicity assay

As a first step towards assessing *in vivo* safety a single dose acute toxicity study was conducted in female Balb/c nude heterozygous mice (>9 weeks of age). Mice were group housed (5 mice/cage) on BioFresh™ bedding (Ferndale, WA), in a temperature and humidity controlled facility (22 °C ± 2 °C) on a 12-hour light:dark cycle (0700 h–1900 h), had free access to food (Prolab^®^ RMH 3000, Purina, Inc., Richmond, IN) and water, and allowed a minimum 7-day acclimatization period. Mice were randomly assigned to four groups (n = 5/group): control (vehicle) and 3 escalating doses (10, 100, 400 mg/kg). 9bWC3 was suspended in 1% carboxymethylcellulose/1% Tween 80 and administered once by oral gavage (10 μL/g body weight). Mice were monitored for 14 days with daily assessments of body weight, food/water consumption, clinical signs, and mortality. Fourteen days post administration, mice were anesthetized with isoflurane, exsanguinated for euthanasia, and gross necropsy performed in a blinded fashion by a laboratory animal medicine veterinarian. This work was approved by the University of Saskatchewan’s Animal Research Ethics Board, and adhered to the Canadian Council on Animal Care guidelines for humane animal use.

### Statistical analysis

For the EDTA assay, SPSS Statistics was used to perform a one-way ANOVA, which identified a statistically significant difference between groups (F(3,14) = 374.102, p = 1.3 × 10^−13^). A Tukey post hoc test revealed statistically significant differences in growth between the phosphopyricin with EDTA treatment (OD_600nm_ = 0.407+/−0.077) and the other three treatment groups: only EDTA (OD_600nm_ = 1.236+/−0.021), only phosphopyricin (OD_600nm_ = 1.4420+/−0.053), and no treatment (OD_600nm_ = 1.260+/−0.037); p < 1.3 × 10^−11^. Levene’s test of homogeneity showed no statistically significant differences between variances across treatments (1.421, p = 0.279).

Statistical evaluation of bactericidal activity of 9bWC2 and 9bWC3 (phosphopyricin) was carried out with SPSS Statistics using the Mann-Whitney test, which indicated statistically significant differences between the control group (supplemented with isopropanol) and both 9bWC2 (U = 0, p < 2 × 10^−6^) and phosphopyricin (U = 0, p < 3 × 10^−7^). The Mann-Whitney test revealed no statistically significant differences in the proportion of food consumption between control mice and those receiving 400 mg/kg phosphopyricin (U = 92, p = 0.78). There were also no statistically significant differences in the proportion of water consumption between those groups (U = 58, p = 0.07), or the change in body weight between day 0 and day 14 (U = 7, p = 0.25).

## Additional Information

**How to cite this article:** Hubick, S. *et al*. A potent synthetic inorganic antibiotic with activity against drug-resistant pathogens. *Sci. Rep.* **7**, 41999; doi: 10.1038/srep41999 (2017).

**Publisher's note:** Springer Nature remains neutral with regard to jurisdictional claims in published maps and institutional affiliations.
